# Thyrotoxic Periodic Paralysis with Thyroid Storm as the First Presentation of Graves’ disease; a Case Report

**DOI:** 10.22037/aaem.v9i1.1083

**Published:** 2021-02-17

**Authors:** Tejaswee Banavathu, Swapnil Tripathi, Pankaj Sukhadiya, Kamlesh Ahari, Durga Shankar Meena, Mahendra Kumar Garg

**Affiliations:** 1 Department of Internal Medicine, All India Institute of Medical Sciences, Jodhpur, India.

**Keywords:** Hypokalemic Periodic Paralysis, Thyrotoxicosis, Graves disease, Quadriplegia, Thyroid Crisis

## Abstract

Thyrotoxic periodic paralysis is a rare endocrine emergency that manifests as acute onset muscle weakness and hypokalaemia secondary to thyrotoxicosis. It mainly occurs due to rapid and dramatic intracellular shift of potassium resulting in hypokalaemia and acute flaccid paralysis. This condition predominantly affects males of Asian descent, and presentation can range from mild generalized weakness to complete quadriplegia, as seen in our case. We herein report a case of a 40-year-old female, who presented to us with acute onset flaccid quadriplegia and thyroid storm, which is the first ever manifestation of previously undiagnosed Grave’s disease. Liver abscess was found to be the underlying trigger for thyrotoxic paralysis and thyroid storm.

## Introduction

Periodic paralysis and thyroid storm are two different manifestations of thyrotoxicosis-related medical emergencies, which are life-threatening without prompt diagnosis and treatment (1, 2). Thyrotoxic periodic paralysis (TPP) is the acquired form of hypokalaemic paralysis, which usually occurs after the second or third decade of life. The incidence of TPP among the Western and Asian population is around 0.1% and 1.8%, respectively (3). Hypokalaemic paralysis with thyrotoxic crisis, as an initial presenting feature of hyperthyroidism, is rarely reported in the literature (4). Our case had both of these features on the first hospital visit along with an occult liver abscess, which was the probable trigger for TPP. Diagnosis of TPP is often overlooked, which may have fatal consequences in the form of cardiac arrhythmias and respiratory paralysis (5). Emergency physicians should have a high index of suspicion of TPP while treating a case of hypokalaemic paralysis.

## Case presentation:

A 40-year-old Asian female presented to the emergency department with complaints of high-grade fever associated with palpitation for 7 days, loose stools for 2 days and altered sensorium for the last 1 day. She had a history of episodic right upper quadrant pain and weight loss for the last 1 month. No history of blood in the stool, cough, chest pain, headache, or vomiting could be elicited. She had not had similar episodes in the past. On presentation, she was drowsy with a Glasgow Coma Score (GCS) of E3V3M4, febrile (temperature: 100.8°F), blood pressure of 98/68 mmHg, pulse rate of 160/minutes, and respiratory rate of 24/minutes. General physical examination revealed a thin-built female with pallor and acral hypopigmentation (present since birth). Neurological examination showed flaccid paralysis of all limbs with power of 1/5 and diminished reflexes. No signs of meningeal irritation were present. No sensory or cranial nerve deficit was detected. Systolic flow murmur was heard on cardiac auscultation suggestive of hyperdynamic circulation. Rest of the systemic examination was unremarkable. Based on the clinical findings, acute transverse myelitis, Guillain-Barre syndrome (GBS) and periodic paralysis were considered as differential diagnoses of acute flaccid paralysis. Magnetic resonance imaging (MRI) of spine and nerve conduction studies were normal, which ruled out acute transverse myelitis and GBS. 

Arterial blood gas analysis revealed serum potassium of 2.23 mmol/L with normal acid-base status. Other blood investigations are tabulated in table 1. Electrocardiogram showed sinus tachycardia with ST segment depression in V3-V6. The initial diagnosis of hypokalaemic paralysis was made, and she was given intravenous potassium supplementation along with intravenous fluids, but her altered sensorium was still unexplained. In addition, computed tomography (CT) of brain and cerebrospinal fluid (CSF) analysis were done, both were found to be normal. On further evaluation, her thyroid function test showed thyroid stimulating hormone (TSH) = 0.0056 mIU/L, T3 = 28.26 pg/ml, T4 = 66.66 ng/ml, TSH Receptor antibody level of 30.27 IU/L, and Anti Thyroperoxidase (TPO) of 1433 IU/L (Table 1). Thyroid scan also showed an increased radioactive iodine uptake. The final diagnosis of Graves’ disease with thyroid storm and thyrotoxic hypokalaemia paralysis was made on the basis of clinical features and blood investigations. 

Patient was immediately put on propylthiouracil (600 mg loading dose followed by 200 mg, 6 hourly), propranolol (80 mg, 8 hourly), and hydrocortisone (100 mg intravenous, 8 hourly) along with supportive care, which included cold water sponging and antipyretics. On investigating the precipitant of thyroid storm, she was found to have an abscess about 90 ml in segment VI of liver. She underwent needle aspiration of the abscess and received intravenous antibiotics (metronidazole). Abscess culture was sterile. In addition, blood and urine culture were also performed to rule out other potential sources of infection, which were sterile.

The patient regained full consciousness on day 3 and a repeat neurological examination revealed quadriparesis with predominant proximal muscle involvement. On day 3, her potassium level was 3.12 mmol/L which increased to 5.4 mmol/L on day 5 without further potassium supplementation suggesting rebound hyperkalaemia. At day 6 of hospitalization, her power of bilateral lower limb and upper limb improved and the patient was able to stand with support and feed herself. Hydrocortisone was stopped on day 6 due to the improvement in thyroid storm. Patient was continued on antibiotics in view of liver abscess for 14 days along with antithyroid drugs. On day 14 the patient was discharged in fully oriented state, with improved power of 4/5 in all limbs. The dose of propylthiouracil was further reduced (100 mg, 8 hourly), which was scheduled for tapering based on further clinical improvement. On day 30 of follow-up, repeat thyroid function showed further improvement (Table 1).

**Table 1:** Laboratory findings of the presented case

**Laboratory Parameters **	**Patient values**	**Normal **
Hemoglobin (g/dl)	11.1	12-15
White blood cell count (/L)	14×10^3^	(4-11) ×10^3^
Serum sodium (mEq/L)	137	135-145
Creatine kinase-MB (U/L)	25	0-24
TSH Receptor Antibody (IU/ml)	30.27	<1.22
Anti TPO antibody (IU/ml)	1433	< 60
Serum calcium (mg/dl)	9.1	8.8-10.6
Serum phosphorus (mg/dl)	3.38	2.5-4.5
Serum magnesium (mg/dl)	1.9	1.5-2.5
Serum anion gap (mmol/L)	8	8-12
**Arterial blood gas analysis**		
pH	7.40	7.35-7.45
pCO2 (mmHg)	23	35-45
HCO3 (mmol/L)	19.6	22-28
Natrium (mmol/L)	140	-
Potassium (mmol/L)	2.25	-
Chloride (mmol/L)	106	-
**Urinalysis**		
pH	7.5	
Urinary Na (mmol/L)	157.1	40-220
Urinary K (mmol/L)	14.47	<20
Urinary Chloride (mmol/L)	121.7	-
Pus cells	Nil	-
Red blood cell	Nil	-
Albumin	Nil	-
**Serum potassium (mEq/L)**		-
On admission	2.23	3.5-5.1
Day 5	5.14	
Day 7	4.2	
**TSH (mIU/L)**		
On admission	0.005	0.3 - 4.0
After 2 weeks	0.016	
At 4 weeks	0.1	
**Free T3** **(pg/ml)**		
On admission	28.26	2.2-4.2
After 2 weeks	3.2	
At 4 weeks	3.82	
**Free T4 (** **ng/dl)**		
On admission	66.66	0.80-1.70
After 2 weeks	3.69	
At 4 weeks	1.20	

TSH: thyroid stimulating hormone; TPO: Thyroperoxidase; Na: natrium; K: potassium.

## Discussion

Neurological involvement in hyperthyroidism is not uncommon and may present with diverse clinical features. Symptoms may vary from mild features like tremors, chorea, headache, peripheral neuropathy, and hyperthyroid myopathy to life-endangering seizures, thyroid storm and thyrotoxic periodic paralysis (6). TPP as the first presenting feature of hyperthyroidism is a rare entity. Another highlight of our report was thyroid storm, which was precipitated by liver abscess along with TPP.

Thyrotoxic periodic paralysis is a medical emergency, manifesting as paraparesis or quadriparesis mainly in the Asian population suffering from hyperthyroidism. It is more commonly present in young males compared to females with a ratio of approximately 20:1 (7). Clinical features of thyrotoxic periodic paralysis include recurrent attacks of muscle weakness with predominant proximal muscle involvement (8). Episodes may range from prodromes (mild weakness, cramps, stiffness) to complete quadriparesis and respiratory muscle involvement (9). Guillain-Barre syndrome, myasthenia gravis, transverse myelitis, snake envenomation and hysterical disorder are among the common differential diagnoses of thyrotoxic hypokalaemic paralysis. Unlike GBS, respiratory involvement is rare in TPP (10). In our patient, the absence of toxin exposure, lack of diurnal variation of weakness, and normal neuroimaging and CSF findings ruled out the other possibilities. 

Proposed pathogenesis behind TPP is related to ion channel defect. Hypokalaemia is due to transcellular shift of potassium ion, which is controlled by Na^+ ^K^+ ^ATPase pump. The activity of Na^+ ^K^+ ^ATPase is mainly influenced by insulin and beta-adrenergic catecholamines. Increased adrenergic state in thyrotoxicosis leads to increased activity of Na^+ ^K^+ ^ATPase, which causes transcellular shift of potassium resulting in the various manifestations of hypokalaemia (3). Hyper-insulinemic state, stress, infections, high carbohydrate diet, beta-2 adrenergic bronchodilators and strenuous physical activity may also act as TPP precipitants. However, precipitating factors could not be identified in nearly 34% of the cases (11). Genetic mutations have been found to increase susceptibility to TPP, the most commonly noted mutation is the one affecting the gene that encodes Kir2.6, an inwardly rectifying potassium channel, which is regulated by thyroid hormone (12). Diagnosis involves hypokalaemia with normal acid base balance associated with suppressed TSH and raised T3 and T4 levels. The potassium levels have shown to be correlated with severity of muscle weakness; however, no correlation is seen with T3 and T4 levels (2).

Management of TPP includes initial supplementation of potassium along with beta-blockers and achievement of euthyroid state using anti-thyroid drugs. Potassium supplementation should be gradual and under continuous potassium monitoring as total body potassium remains the same, which can cause life-threatening rebound hyperkalaemia after the achievement of euthyroid state. Rebound hyperkalaemia is reported in around 50% of the cases with TPP (13). Beta blockers (propranolol) have 2 mechanisms in TPP, the first one is to antagonise the adrenergic stimulation of Na^+ ^K^+ ^ATPase and the second one is to decrease the peripheral conversion of T4 to T3, which is the active form. Another important facet of TPP management is to mitigate the risk of paradoxical hypokalaemia, which can be seen in 25% of the patients, particularly those with late administration of anti-thyroid drugs and beta blockers (13). Our patient also had simultaneous thyroid storm with thyrotoxic periodic paralysis. Corticosteroids are useful for thyroid storm management but at the same time could precipitate TPP, which makes it even more difficult to treat. Furthermore, our patient also had a liver abscess, which is an unusual trigger for thyrotoxic crisis and periodic paralysis. Further large-scale studies will be needed to understand the effect of corticosteroids on the course of periodic paralysis.

## Conclusion:

Prompt diagnosis and treatment of thyrotoxic periodic paralysis is vital to prevent fatal complications like cardiac arrhythmias. Thyroid function test should be sought in every patient presenting to emergency department with hypokalaemic paralysis. 

## Source of Funding

 None

## Conflict of interest

The authors declare that they have no conflict of interest

## Authors' contributions

All the authors meet the standard criteria of authorship based on recommendations of the international committee of medical journal editors.

## Ethical considerations

Written informed consent was taken from the patient. 
